# Case Report: Anaphylactic shock and ST-elevation myocardial infarction following a bee sting: two deadly diseases in a patient with Kounis syndrome

**DOI:** 10.3389/fcvm.2025.1530829

**Published:** 2025-05-12

**Authors:** Guido Del Monaco, Carolina Pascucci, Federica Catapano, Giulio G. Stefanini, Giuseppe Ferrante

**Affiliations:** ^1^ Department of Cardiovascular Medicine, Humanitas Research Hospital-IRCCS, Rozzano, Milan, Italy; ^2^ Department of Biomedical Sciences, Humanitas University, Pieve Emanuele, Milan, Italy; ^3^ Department of Radiology, Cardiovascular Imaging Unit, Humanitas Research Hospital-IRCCS, Rozzano, Italy

**Keywords:** Kounis syndrome, acute myocardial infarction, thrombosis, anaphylaxis, percutaneous coronary intervention

## Abstract

Kounis syndrome is an acute coronary syndrome occurring in the setting of an allergic reaction, usually caused by drug administration, food ingestion, or insect sting. We report the case of an elderly woman who presented to the emergency room suffering from an anaphylactic shock caused by a bee sting and who was diagnosed with an anterolateral ST-elevation myocardial infarction (STEMI) with moderately impaired left ventricular ejection. The patient was successfully managed with the administration of intravenous antihistaminic drugs and steroids, intravenous fluid volume resuscitation, and intramuscular epinephrine. The patient then underwent emergency coronary angiography, which showed a thrombotic subtotal occlusion of the proximal left anterior descending artery (LAD) and occlusion of the very distal apical LAD due to a spontaneous embolism. This was treated by primary percutaneous coronary intervention with thrombus aspiration and drug-eluting stent implantation in the proximal LAD, achieving a good angiographic result. Nevertheless, on day 3, the patient developed a left ventricular apical thrombosis, as assessed by cardiac magnetic resonance, requiring oral anticoagulation with rivaroxaban, de-escalation of dual antiplatelet therapy from ticagrelor to clopidogrel with acetylsalicylic acid, and finally a switch to dual antithrombotic therapy. The 3-month follow-up was uneventful. This case highlights the importance of prompt identification of Kounis syndrome in patients presenting with severe allergic reactions to allow for the timely implementation of appropriate reperfusion strategies in such high-risk patients with STEMI.

## Introduction

Kounis syndrome (KS), also known as “allergic angina,” is an acute coronary syndrome (ACS) occurring in the setting of an allergic, anaphylactoid, or anaphylactic reaction and was first described in 1991 by Kounis and Zavras (1). KS can be triggered by various stimuli such as drugs, food, and insect stings, which ultimately cause mast cell degranulation, activation of the inflammatory cascade, subsequent vasospasm, and/or coronary plaque rupture or erosion with thrombosis (2). The incidence of KS is likely underestimated, and it has been reported to be more frequent among men (75%), with the age at onset ranging from 40 to 70 years (3). Chest pain is the most frequent symptom on admission (4). The diagnosis of ACS in relation to KS requires clinical suspicion to avoid delay in the implementation of guideline-directed therapies, and the treatment of KS may be challenging because the pharmacological interventions used to manage the allergic reaction may exacerbate coronary vasospasm (5) and reduce coronary blood flow.

Herein, we report the case of an elderly woman who experienced a severe form of KS following a bee sting.

### Patient information

A 75-year-old woman with a history of arterial hypertension, dyslipidemia, obstructive sleep apnea syndrome, epilepsy, and multiple allergies to insects, penicillin, and iodinated contrast agent presented to our emergency room due to a sudden onset of general malaise following a bee sting on her left hand. The patient had a history of previous anterolateral ST-elevation myocardial infarction (STEMI) that was treated with percutaneous coronary intervention (PCI) with drug-eluting stent (DES) implantation to the first diagonal branch (D1) years earlier, and left ventricular systolic function was preserved. The patient was on aspirin (100 mg daily), atorvastatin (40 mg daily), ramipril (2.5 mg daily), and levetiracetam (500 mg twice daily). Cardiological follow-up was regular and uneventful.

### Clinical findings and diagnostic assessment

On admission, the patient was drowsy, albeit responsive to verbal and pain stimuli (Glasgow Coma Scale = 14), complaining of shortness of breath at rest with a respiratory rate of 22 and oxygen saturation of 94% in room air. Her blood pressure was 70/40 mmHg, her heart rate was 110 bpm, and a large maculopapular rash was present on her trunk, arms, and limbs. She was complaining of nausea, had an episode of gastric vomiting, and was suffering from mild epigastric pain. The physical examination found normal cardiac auscultation and a diffuse reduction in lung sounds accompanied by bilateral basal rales. The arterial blood gas analysis (Table 1, upper panel) indicated type I respiratory failure coupled with metabolic lactic acidosis. Blood examinations showed blood cell count, electrolytes, renal and hepatic function, and high-sensitivity troponin I (hs-TnI) to be within normal limits (Table 1, lower panel). The patient received low-flow oxygen therapy, intravenous hydrocortisone (200 mg) and chlorphenamine (10 mg), and a fluid bolus of 500 ml saline, achieving a partial resolution of the skin rash, although no significant improvement in blood pressure occurred, and the remaining symptoms persisted. Therefore, intramuscular epinephrine (1 mg) was administered, with immediate recovery of blood pressure and improvement in her neurological status.

**Table 1:** Arterial blood gas analysis and blood examinations at admission.

Arterial blood gas analysis		
Variable	Value	Reference interval
pH	7.33	7.35–7.45
pCO_2_ (mmHg)	36	35–45
pO_2_ (mmHg)	58	>60 mmHg
Na+ (mmol/L)	135	135–145
K+ (mmol/L)	3.3	3.5–5
Hb (g/dl)	13.3	>12
HCO_3_^−^ (mmol/L)	19	22–28
Lactate (mmol/L)	4.8	<2
BE (mmol/L)	−6.9	+2/−2
Glucose (mg/dl)	332	70–99
Blood examinations		
Variable	Value	Reference interval
AST (IU/L)	17	<35
ALT (IU/L)	9	<35
Bilirubin (mg/dl)	0.9	0.3–1.2
Hs-TnI (ng/L)	8.1	2.2–11.6
CPK (IU/L)	52	15–145
Mgb (mcg/L)	89	14–65
BNP (ng/L)	54	<100
Urea (mg/dl)	34	17–43
Creatinine (mg/dl)	0.92	0.5–1
Na (mmol/L)	140	135–145
K (mmol/L)	3.4	3.5–5
WBC (10^9^/L)	11.58	4–10
Hb (g/dl)	13	12–16
PLT (10^9^/L)	477	150–450
CRP (mg/dl)	1.2	<0.5
Glucose (mg/dl)	212	70–99

pCO_2_, partial pressure of carbon dioxide; pO_2_, partial pressure of oxygen; Na+, sodium; K+, potassium; Hb, hemoglobin; HCO_3_^−^, bicarbonate; BE, base excess; AST, aspartate aminotransferase; ALT, alanine aminotransferase; hs-TnI, high-sensitivity troponin I; CPK, creatine phosphokinase; Mgb, myoglobin; BNP, B-type natriuretic peptide; Na, sodium; K, potassium; WBC, white blood cells; Hb, hemoglobin; PLT, platelets; CRP, C-reactive protein.

**Figure 1:**
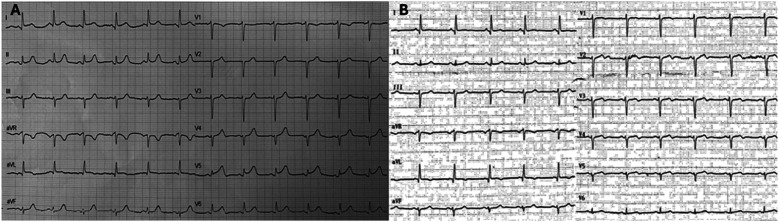
Baseline and discharge ECGs. **(A)** ECG at admission: sinus rhythm with HR∼75 bpm, normal AV and IV conduction, mild left axis deviation with 1.5 mm ST-segment elevation in leads V5 and V6, and q waves in leads I and aVL. **(B)** ECG at discharge: sinus rhythm with HR∼75 bpm, normal AV and IV conduction, mild left axis deviation with ST segment elevation resolution, and biphasic T waves in leads V2–V5 with persistent q waves in leads I and aVL. ECG, electrocardiogram; HR, heart rate; AV, atrio-ventricular; IV, intra-ventricular.

**Figure 2:**
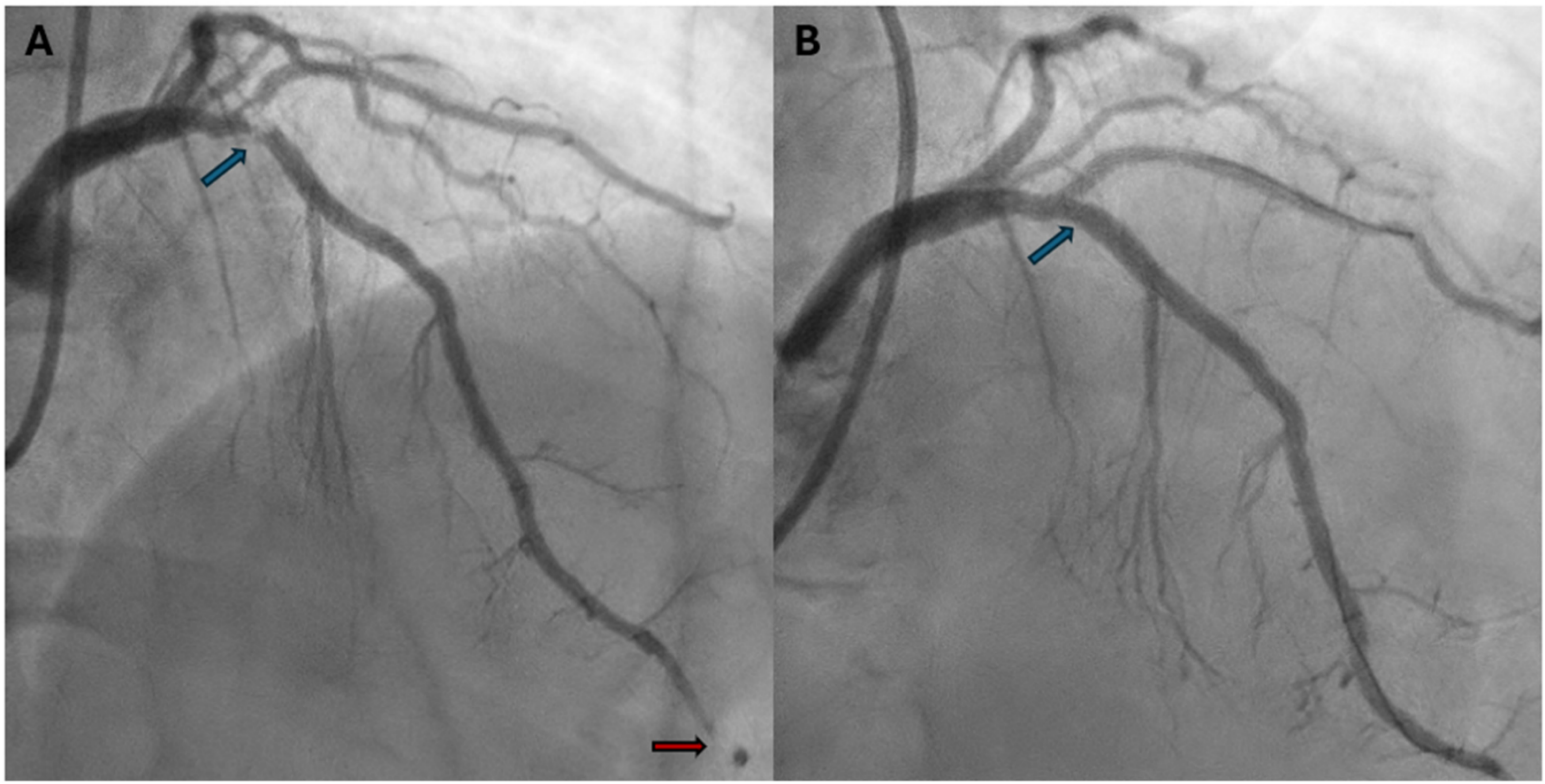
Baseline and final coronary angiography. **(A)** Baseline coronary angiography (RAO 30°, cranial 30°) shows a thrombotic subocclusion of proximal LAD with ostial involvement of D1 (blue arrow). The previously implanted DES on D1 is patent and free of restenosis. A perfusion defect in the distal LAD suggests spontaneous distal embolization (red arrow). **(B)** Final coronary angiography (RAO 30°, cranial 40°) shows a good angiographic result after PCI to LAD-D1 bifurcation with single DES implantation (3.5 × 18 mm) to the LAD, a kissing balloon with NC 3.0/2.0 mm to LAD/D1, and the proximal optimization technique with a 3.5 mm NC balloon (blue arrow). RAO, right anterior oblique; LAD, left anterior descending; D1, first diagonal; DES, drug-eluting stent; PCI, percutaneous coronary intervention; NC, non-compliant balloon.

**Figure 3:**
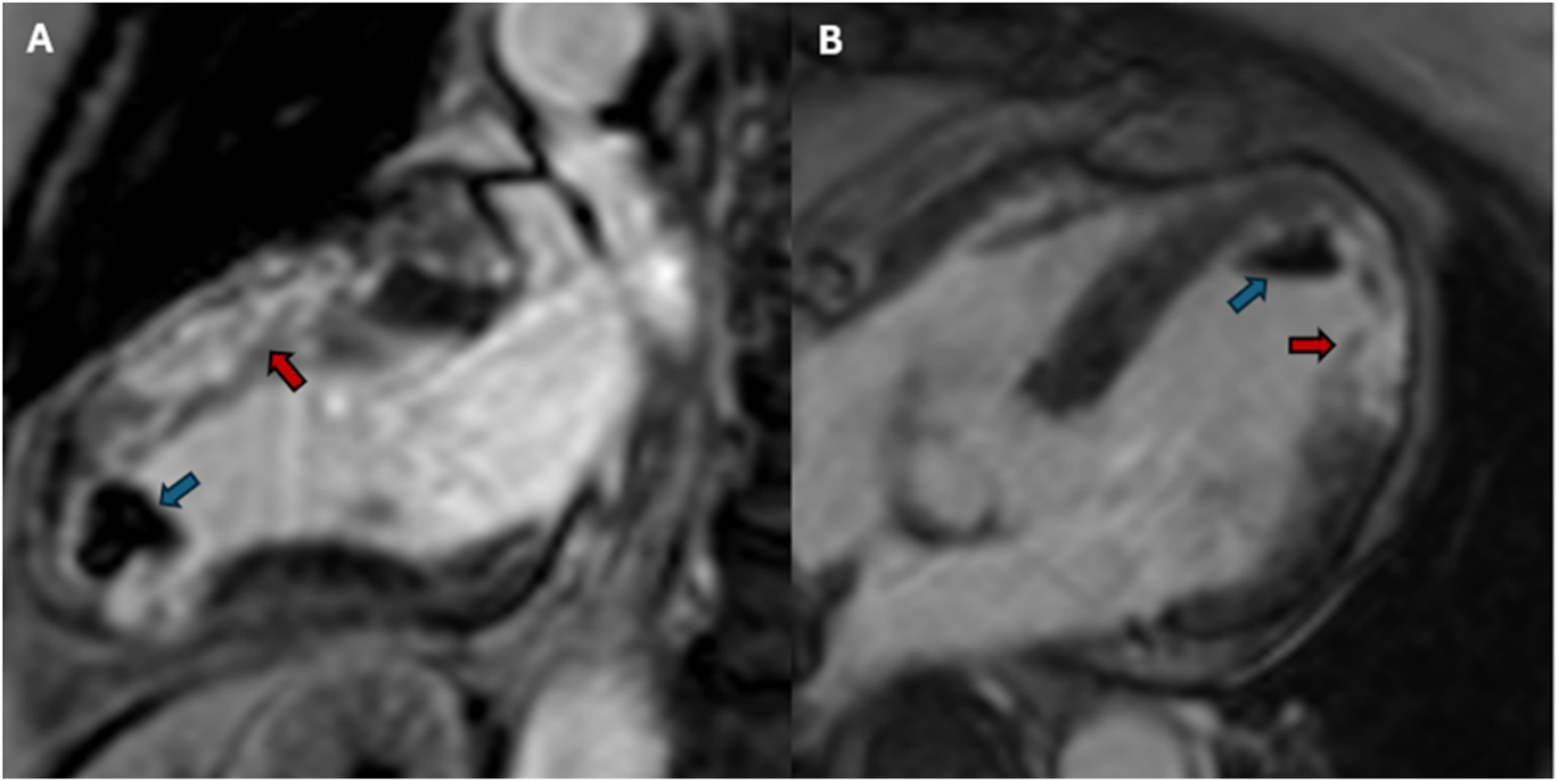
Post-PCI cardiac magnetic resonance imaging. **(A)** Post-PCI cardiac magnetic resonance 2-chamber view. LGE sequences showing an extensive transmural ischemic scar in the anterior wall (red arrow) and a large left ventricular apical thrombus (blue arrow). **(B)** Post-PCI cardiac magnetic resonance 4-chamber view. LGE sequences showing an extensive transmural ischemic scar in anterolateral wall (red arrow) and a large left ventricular apical thrombus (blue arrow). PCI, percutaneous coronary intervention; LGE, late gadolinium enhancement.

Due to the persistence of epigastric pain, a 12-lead electrocardiogram (ECG) was performed (Figure 1A), showing sinus rhythm at 75 bpm with 1.5 mm ST-segment elevation in leads V5 and V6. Bedside echocardiography showed a mild reduction of left ventricular ejection fraction (LVEF) of 45%, left ventricular apical akinesia, and hypokinesia of the mid-to-distal anterior and anterolateral segments. No significant valvular abnormalities or pericardial effusion were found. A diagnosis of STEMI was made, and the patient received an aspirin loading dose of 250 mg and ticagrelor of 180 mg. Emergency coronary angiography showed a thrombotic sub-occlusion of the left anterior descending artery (LAD) involving the ostium of D1, patency of the previously implanted stent to D1 without restenosis (Figure 2A), and a spontaneous distal embolization defect of the apical LAD with thrombolysis in myocardial infarction (TIMI) 1 flow. Manual thrombus aspiration was performed to the LAD lesion at the level of the bifurcation with D1 only. It was not attempted at the very distal apical LAD because of the small caliber of the vessel. A residual tight stenosis of the LAD at the bifurcation level was treated with a 3.5 × 18 mm DES implantation to the LAD, kissing balloon inflation (3.0/2.0 mm) to LAD-D1, and the final proximal optimization technique with a 3.5 mm NC balloon. Post-procedure echocardiography showed an LVEF of 40%, with akinesia of the apex and of the mid-to-distal anterior and anterolateral segments, without evidence of mechanical complications. Blood examinations showed a peak value of hs-TnI of 19.980 ng/L at 32 h. Continuous ECG monitoring recorded frequent premature ventricular contractions and a brief episode of non-sustained ventricular tachycardia. Therefore, beta-blocker therapy with intravenous metoprolol was started. However, shortly after drug administration, the patient experienced a transient episode of epigastric pain that was responsive to sublingual nitroglycerin. In suspicion of drug-induced vasospasm, metoprolol was promptly discontinued with no symptom recurrence. Heart failure medications including an ace-inhibitor, a mineralocorticoid receptor antagonist, and a sodium-glucose co-transporter 2 inhibitor were started and well-tolerated. On day 3 following the index procedure, cardiac magnetic resonance imaging showed a moderate LVEF reduction (38%), akinesia in the anterior and anterolateral mid-apical regions with apical thrombosis (9 × 20 mm), and late gadolinium enhancement (LGE) extension wall thickness >75% in the same regions with wall motion abnormalities (Figures 3A,B). Rivaroxaban at 20 mg daily was started and ticagrelor was discontinued and de-escalated to clopidogrel, which was continued for 1 week after the index procedure. On day 7, the patient was discharged home asymptomatic on dual antithrombotic therapy with aspirin 100 mg daily plus rivaroxaban 20 mg daily. Discharge ECG showed a reduced R wave progression on the anterior leads with resolution of the ST-segment elevation (Figure 1B). At the 3-month follow-up, the patient was free from angina. She is on dual anti-thrombotic therapy with aspirin and rivaroxaban. A cardiac magnetic resonance found an LVEF of 42%, an unchanged LGE pattern, and a significant reduction in LV apical thrombus size (2 × 8 mm).

## Discussion

KS is a rare and underrecognized cause of ACS and is associated with high morbidity and mortality. According to prior studies, KS may not be just a single-organ disease, but a complex multisystem and multi-organ arterial condition, affecting other arterial districts (mesenteric and cerebral) (6, 7). Risk factors for KS include arterial hypertension, dyslipidemia, cigarette smoking, and a history of previous allergic reactions (8). KS can be triggered by several factors, such as, most frequently, drugs (mainly antibiotics), food, and insect bites (4).

Mast cell degranulation releases vasoactive and pro-inflammatory mediators—such as histamine, leukotrienes, and proteases—which can trigger coronary vasospasm, increase vascular permeability, and promote plaque rupture or erosion, leading to thrombus formation (2).

Clinical presentation includes cardiac symptoms (chest pain, dyspnea, and arrhythmias) in the setting of overt allergic/anaphylactic reactions, which can ultimately present as systemic hypotension, shock, and/or cardiac arrest. Notably, cardiac symptoms occur in the first hour after exposure to the allergic agent in approximately 80% of cases (1).

KS can be classified into three subtypes according to its pathophysiological mechanisms: type I (the most frequent), consisting of coronary vasospasm in the absence of overt coronary artery disease; type II, caused by plaque rupture or erosion with superimposed thrombosis, and type III, the rarest form, occurring in the presence of a pre-existing coronary stent, which can be further categorized as type III-A (stent thrombosis) or type III-B (in-stent restenosis) (9, 10). Giovannini et al. have recently introduced KS type IV, which is characterized by coronary artery bypass graft thrombosis (11). Regardless of the classification, all KS subtypes share a common initial mechanism: an allergic reaction that triggers mast cell degranulation and activates the inflammatory cascade, which may affect coronary blood flow (12).

We report a case of type II KS presenting with STEMI and angiographic evidence of a high thrombus burden likely to have been caused by plaque destabilization to the proximal LAD, and not affecting the previously implanted stent to D1. The use of intravascular imaging, such as optical coherence tomography and, to some extent, high-resolution intravascular ultrasound, may have allowed the identification of the specific coronary pathology, i.e., rupture vs. erosion (13), underlying the coronary thrombosis and the histological analysis of the coronary aspirate may be useful for detecting the presence of eosinophils and mast cells inside the thrombus, as previously reported (14).

Furthermore, intravascular imaging guided-PCI is associated with improved clinical outcomes in complex PCI, particularly for bifurcated lesions (15). Of note, the coexistence of ST-segment elevation in anterior leads and apical akinesia in echocardiography may prompt a differential diagnosis of takotsubo syndrome. Indeed, the association between takotsubo syndrome and KS has been defined as the “ATAK complex” (adrenaline, takotsubo, anaphylaxis, and Kounis) (16) and several case reports have reported the complex and challenging nature of this disease in clinical practice (17, 18). Notably, cardiac magnetic resonance imaging is an accurate imaging technique to distinguish between takotsubo syndrome and STEMI in unclear ECG patterns and allows for the assessment of the infarct size, microvascular obstruction, and complications such as left ventricular apical thrombosis following acute myocardial infarction (19, 20).

There is no formal consensus for the management of KS. Nevertheless, prior case series have highlighted the importance of simultaneous optimal treatment of the ongoing allergic reaction and the implementation of guideline-directed treatment for ACS (21). In line with these recommendations, our patient received an optimal management regimen for the allergic reaction and underwent primary PCI. Intravenous antihistamine drugs and short-acting corticosteroids, an intravenous fluid bolus, and intramuscular epinephrine were administered. Antihistamine drugs are the mainstay treatment of allergic reactions, but they have been reported to be associated with the occurrence of hypotension and worsening of coronary perfusion (22, 23). Corticosteroids appear to be safe in KS, however, it has been suggested that they could increase the risk of cardiac aneurysm and free wall rupture in patients with STEMI (24). With respect to intravenous fluid bolus and intramuscular adrenaline administration, these pharmacological interventions play a pivotal role in patients presenting with anaphylactic shock, as adequate intravascular volume expansion works synergistically with adrenaline to prevent the deterioration of the hemodynamics toward refractory shock and cardiac arrest. Nevertheless, the use of intravenous epinephrine requires careful dosing as it may worsen coronary vasospasm and it may increase the risk of ventricular arrythmias (16, 25, 26). With respect to coronary vasospasm in patients with KS, additional medications such as beta-blockers need to be used with caution (27). Nitrates and calcium channel blockers, particularly diltiazem or verapamil, are effective in preventing and terminating episodes of angina due to vasospasm. However, an optimal dosage of these drugs should be tailored according to systemic blood pressure, and caution is needed when using diltiazem or verapamil in patients with left ventricular systolic dysfunction (28).

With respect to the optimal antithrombotic therapy for the management of left ventricular apical thrombosis, there is no clear evidence from randomized clinical trials about the superiority of direct oral anticoagulants vs. vitamin K antagonists. The selection of the most appropriate drug class should take into account the patient’s bleeding risk, given the presence of simultaneous single or dual antiplatelet therapy. In our case, we selected rivaroxaban in line with most recent evidence (29).

We acknowledge a number of limitations. First, we did not measure the blood levels of histamine, IgE, and tryptase. Second, we did not assess the histological composition of the thrombotic material retrieved with the thrombectomy device.

## Conclusions

STEMI may occur in the setting of anaphylactic shock and may represent the clinical presentation of KS. Prompt recognition of coronary complications of allergic reactions and timely evidence-based treatment of both the allergic reaction and the ACS is mandatory to improve the patient’s clinical outcome.

## Data Availability

The original contributions presented in the study are included in the article/Supplementary Material, further inquiries can be directed to the corresponding author.
